# Patterns and Perceptions of Climate Change in a Biodiversity Conservation Hotspot

**DOI:** 10.1371/journal.pone.0032408

**Published:** 2012-02-27

**Authors:** Joel Hartter, Mary D. Stampone, Sadie J. Ryan, Karen Kirner, Colin A. Chapman, Abraham Goldman

**Affiliations:** 1 Department of Geography, University of New Hampshire, Durham, New Hampshire, United States of America; 2 Department of Environmental and Forest Biology, College of Environmental Science and Forestry, State University of New York College of Environmental Science and Forestry, Syracuse, New York, United States of America; 3 National Center for Ecological Analysis and Synthesis, University of California Santa Barbara, Santa Barbara, California, United States of America; 4 Department of Anthropology, University of Florida, Gainesville, Florida, United States of America; 5 Department of Anthropology & McGill School of Environment, McGill University, Montreal, Canada; 6 Wildlife Conservation Society, Bronx, New York, United States of America; 7 Department of Geography, University of Florida, Gainesville, Florida, United States of America; University of Oxford, United Kingdom

## Abstract

Quantifying local people's perceptions to climate change, and their assessments of which changes matter, is fundamental to addressing the dual challenge of land conservation and poverty alleviation in densely populated tropical regions To develop appropriate policies and responses, it will be important not only to anticipate the nature of expected changes, but also how they are perceived, interpreted and adapted to by local residents. The Albertine Rift region in East Africa is one of the world's most threatened biodiversity hotspots due to dense smallholder agriculture, high levels of land and resource pressures, and habitat loss and conversion. Results of three separate household surveys conducted in the vicinity of Kibale National Park during the late 2000s indicate that farmers are concerned with variable precipitation. Many survey respondents reported that conditions are drier and rainfall timing is becoming less predictable. Analysis of daily rainfall data for the climate normal period 1981 to 2010 indicates that total rainfall both within and across seasons has not changed significantly, although the timing and transitions of seasons has been highly variable. Results of rainfall data analysis also indicate significant changes in the intra-seasonal rainfall distribution, including longer dry periods within rainy seasons, which may contribute to the perceived decrease in rainfall and can compromise food security. Our results highlight the need for fine-scale climate information to assist agro-ecological communities in developing effective adaptive management.

## Introduction

Understanding local people's perceptions of climate change is fundamental to addressing the dual challenge of land conservation and poverty alleviation in densely populated tropical regions [1]. Tropical deforestation is a major cause of land degradation with impacts on local biodiversity and projected impacts on climate change. In tropical forested areas, protected areas (PAs) are generally smaller, scarcer, and more threatened than savannah PAs [2]. Domesticated landscapes outside these PAs are important because they represent reservoirs of land, resources, and economic opportunity for people [3], [4]. Increased population density leads to increased land conversion and intensification surrounding PAs [5], which leads to altered ecological function and potentially the loss of biodiversity within PAs [6]–[12]. For local people in most of the tropics, the main climatic issue is not temperature change, which varies little seasonally, but rather variable precipitation. Changes in precipitation quantity and pattern will impact the productivity of both tropical forests and neighboring agricultural lands. People are expected to alter their farming practices in response, often by increasing their cultivated areas and/or cultivating land more frequently and intensively – often termed “extensification” and “intensification” respectively.

The primary mechanism used to protect remaining tropical forest biodiversity is PA establishment [13], [14], particularly in regions with high human densities [15], [16]. A major management concern is that PAs will not be resilient to population pressure and land use intensification and extensification outside their boundaries [17]. Climate change will further exacerbate pressures on PAs as they decline in habitat suitability for the species they protect [18]–[22]. This juxtaposition of biodiversity preservation and intensification/extensification to sustain rural livelihoods greatly challenges both the intentions of conservation infrastructure (PAs, corridors, e.g.) and poverty management and alleviation [1], [23].

The Albertine Rift region in East Africa (313 km^2^) is one of the world's most threatened biodiversity hotspots [24]–[27]. This region, known for its extremely high species richness and endemism, has more vertebrate species and more endemic and threatened vertebrate species than anywhere else in Africa [24], [27], [28]. In Uganda alone, there are 21 mammals, 12 birds, 54 fish, and 38 invertebrates listed as endangered by the International Union for Conservation of Nature (IUCN). This area is highly threatened by habitat loss, where there is also a high demand by local people for land and natural resources to support intensive smallholder agriculture [29]. Thus, conservation of remaining forested areas is a high priority [30]. PA managers and conservation groups are particularly concerned about the impacts of climate variability and change on regional resource availability [16], [31] given the potential impacts on wildlife [32], [33].

This region has garnered international attention primarily due to its key position in biodiversity conservation, but it also maintains some of the fastest growing and densest rural human populations in the world. The Ugandan population continues to grow exponentially at an estimated 3.3% (2005–2010), which ranks eighth highest in the world [34]. More alarming is that Uganda has the second youngest population in the world, with almost 49% under 15 years old [35]. With few mineral resources, agricultural products are the main economic resource in Uganda, where over 80% of the land is used for small-scale farming and nearly 80% of the population are farmers [36]. Livelihoods of rural populations depend on rain-fed agriculture and locally-derived natural resources. This makes them very sensitive to variability in the amount and timing of seasonal rainfall. Delayed, decreased, and even increased rainfall can impact crop productivity [37].

Knowledge of rainfall variability and its temporal and spatial patterns is essential for food security, water resource and land use management. This knowledge is based on a longstanding experience and familiarity with seasonal patterns of rainfall and a set of local climate indicators (e.g., presence, absence, and direction of winds; humidity; clouds) that provide clues of season onset and cessation [38]–[40]. Therefore, perceptions of climate change may vary based on the number of years spent as a farmer, amount of formal education, wealth, gender, and age [41]–[43]. Elsewhere, observed changes in rainfall variability is thought to be a higher threat to rain-fed agriculture, and thus rural livelihoods, than changes in total rainfall [44]. In addition, climate-induced changes in food abundance within PAs may cause wildlife to seek alternate food sources, making farms near the PA boundary more vulnerable to crop damage and livestock predation [45], [46]. The lack of long-term, high-frequency instrumental climate records for this region has made characterizing this variability, and the impacts on human livelihoods, difficult [47].

Much of the attention on climate change in Africa has been focused on the more dramatic impacts in drier areas [48], shifting attention away from wetter areas, such as the Albertine Rift. The spatial and temporal patterns of rainfall in the Albertine Rift are highly variable [49], [50] due to complex topography, large inland water bodies, and the existence of large tracts of forest [51], [52]. Previous regional- and continental-scale characterizations of East Africa rainfall variability [51], [53]–[55] indicate that total annual rainfall has increased since the 1980s over much of East Africa [53]. However, these studies appear to run contrary to reports by farmers in western Uganda, who cite changes in weather patterns, including increasing temperatures and decreasing rainfall, as a cause of decreased crop yields [56]. Many have reported drought-stressed plants and the loss of entire seasons' worth of crops that either died or did not grow, forcing many rural households to purchase food, move, or go hungry. Such inconsistencies between regional studies and local perceptions underscore the need for more local-scale information on rainfall season onset, cessation, frequency, and intensity to assist agro-ecological communities in developing effective adaptive management at relevant and appropriate temporal scales [49], [54], [57]–[59].

**Figure 1:**
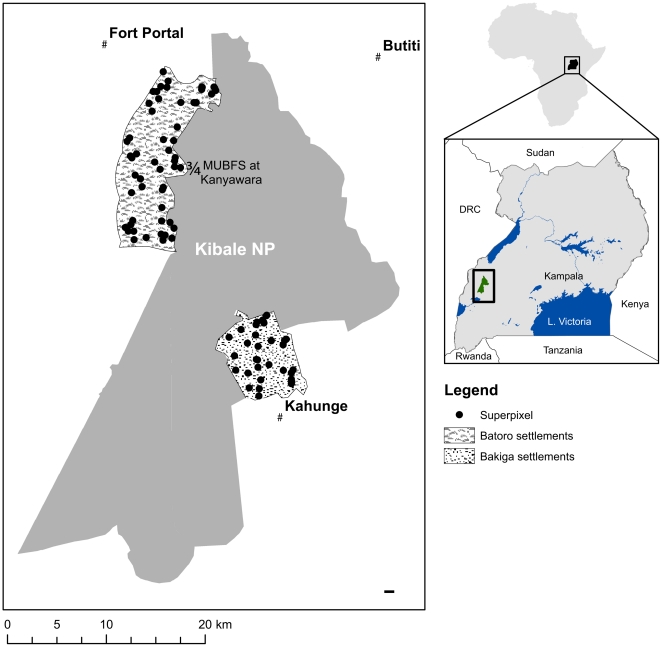
Kibale National Park and locations where household interviews were conducted.

In this study, we couple an analysis of local-scale rainfall variability with survey data collected in densely populated communities surrounding Kibale National Park (795 km^2^, Fig. 1), a forest park in western Uganda. Recent daily rainfall data from within Kibale were used to define rainy and dry seasons and quantify rainfall variability to provide a context for validating local perceptions of changes in seasonal and intra-seasonal rainfall and variability. We hypothesize that there are differences in the perceptions of local people, and that these perceptions vary by residence time, household location and demographics (e.g., wealth and gender) per the results of previous surveys regarding resource use and park impacts conducted by Hartter and Goldman [2], [60].

## Methods

Understanding local trends in rainfall is very important for farmers growing subsistence and cash crops [59], [61]–[64] in order for them to develop coping mechanisms. For many regions in Africa, the fine-scale environmental and social information required to assist agro-ecological communities in developing effective climate change adaptation management practices is scarce or non-existent. Particularly, the lack of daily instrumental weather records representing rainfall over western Uganda [47] has limited previous investigations of inter-annual rainfall variability. Thus we present a rare analysis of available daily rainfall data as a context for understanding how local farmers' perceptions of climatic change impact their decisions concerning land and resource use.

### Study Area

Kibale National Park in western Uganda is a remnant of a transitional forest between savannah and mid-altitude tropical forest surrounded by a large agricultural population. Kibale was originally demarcated in 1932 as a Crown Forest Reserve and changed to a national park in 1993 [29], [65]. It protects 12 species of primates (including the endangered chimpanzee, *Pan troglodytes*), making it one of the most diverse primate communities in the world. Kibale lies near the transition between wet equatorial and moist subtropical precipitation regimes. Elevation increases across the study area from around 900 m in the east to >5000 m toward the Rwenzori Mountains along the western border of Uganda and the Democratic Republic of Congo. This generates a diverse landscape of variable topography, disparate and discontinuous land cover types, and seasonally distinct forcings on weather patterns, all of which may influence the distribution of rainfall at fine spatial and temporal scales. The average annual rainfall at Kibale (at Makerere University Biological Field Station, MUBFS) is 1,654 mm (1968–2010) (standard deviation = 196 mm). However, the seasonal distribution of rainfall varies significantly in response to the north-south migration of the Intertropical Convergence Zone (ITCZ), which results in a bi-modal rainfall pattern consisting of two rainy seasons separated by two dry seasons. Seasons are defined as: 1) first dry: approximately early December through late February; 2) short rains: approximately early March through mid-to-late May; 3) second dry: approximately late May through early September; 4) long rains: September through November. Elevation and proximity to large bodies of water account for some sub-regional and sub-seasonal rainfall variability [49]–[51], [54].

Kibale is situated in one of the most densely populated areas in Sub-Saharan Africa [66]. The population around Kibale has increased over 300% between 1959 and 1990 [45]. In 2006, the population density within 5 km of the park boundary was estimated to be over 260 individuals/km^2^ [60], ranging as high as over 600 individuals/km^2^ (C. MacKenzie, unpublished data). Almost 95% of the population in this area, predominately Batoro and Bakiga tribes, subsists through farming and uses firewood as the primary energy source for cooking [67]. Farms are relatively small, with most less than 5 hectares. Farmers plant more than 20 species of subsistence crops in two cropping cycles. Main staple foods are cooking bananas, sweet potatoes, Irish potatoes, beans, groundnuts, maize, and cassava.

### Rainfall Data

Given the bi-modal pattern of intra-annual rainfall, total annual rainfall is not a useful metric for identifying inter-annual variability and trends in rainfall at practical temporal scales [50]. Due to the lack of daily rainfall records within western Uganda, previous studies estimated total seasonal rainfall using the sum of total monthly rainfall for the months corresponding to the occurrence of each season [49], [50], [54], [68]. While national and regional-scale studies of East African rainfall are limited to monthly rainfall observations, daily rainfall is available for Kibale at MUBFS for the climate normal period 1981 to 2010. This dataset, which is over 90% complete, represents the only long-term record of daily rainfall for Kibale and surrounding area that we are aware of. These data were used to:

1. identify season onset (ONS), cessation (CES), duration (DUR), and total seasonal rainfall (*P*) [58], [69];
2. estimate the number of days in each season with rainfall (RD), reported as percent of season, and the average daily rainfall, or intensity (INT), for rain days within each season [58], [69];
3. identify trends in inter-annual variance about the mean for and dependence between seasonal rainfall and season onset, cessation, duration, and intensity;
4. compute the Standardized Precipitation Index (SPI) as a measure of the relative “dryness” of each season between 1981 and 2010 [70]–[73].

The recommended minimum value for measured total daily rainfall in determining a “rain” or “no rain” day varies by region from 0.30 to 1.00 mm [74], [75]. To be conservative, a daily rainfall threshold for a rain day of greater than or equal to 1.00 mm was used here. Application of cumulative daily rainfall statistics to the Kibale dataset was insufficient for delineating rainy season onset because three or more consecutive days with heavy rainfall are common during the dry seasons. Changes in the amount and distribution of seasonal rainfall occur during the dry seasons as well. Therefore, a model that approximates both rainy and dry season onset was developed using the duration, or persistence, of dry periods identified as cumulative days without rainfall and applied here to predict season onset. Although the cumulative rainfall amount over the transition between seasons is highly variable, a change in the number of consecutive days without rainfall was observed at the onset of both rainy and dry seasons.

Season onset estimates were based on daily rainfall patterns and compared to onset predictions based on the cumulative days without rain followed by cumulative rainfall amounts. Rainy season onset is the first of two or more consecutive rain days followed by three or fewer consecutive days with no rain. A multiday total rainfall of 20 mm or greater over the first multi-day period with rain is used to distinguish between a rainy season onset and a rainy period within a dry season. Dry season onset is similarly defined as the first of five or more consecutive days with no rain followed by four or fewer consecutive rain days. In the case of dry season onset, a multi-day total rainfall of less than 20 mm over the transition from rain to dry season is used. Trends in rainfall variables and correlations between rainfall season statistics exceeding the 90% confidence levels (c.l.) are considered significant. Statistics exceeding the 95% and 99% c.l. are also identified [76].

### Standardized Precipitation Index (SPI)

The original SPI [71] is a drought index that categorizes rainfall over a specified period of time (e.g., long rains in 1987) as above, below or within the range of normal variability based on the average and standard deviation for the time period over the entire dataset (e.g., total rainfall for all long rains from 1981–2010). SPI has been shown to be useful in tropical forested regions [70], [73]. Values between −1.0 and +1.0 are within the range of normal variability (e.g., near normal), meaning that the total seasonal rainfall is within one standard deviation of the 1981–2010 mean season rainfall [71], [77]. Seasons with SPI values greater than 1.0 indicate total season rainfall one or more standard deviations above the mean season rainfall (i.e., moderately, very, or extremely wet seasons). Seasons with SPI values less than −1.0 are seasons in which the total rainfall was one or more standard deviations below mean season rainfall (i.e., moderately, very, or extremely dry seasons).

SPI was calculated for the total seasonal rainfall to identify the relative variability about the seasonal mean for the period of record. The seasonal time series was first fitted to a gamma distribution, Γ(*α*), from which a cumulative probability function of Γ(*α*) was calculated [50], [71], [73]. The probability density function of Γ(*α*) was transformed resulting in SPI values for each season that are a set of standard normal random variables with a mean of zero and variance of one [71], [72]. A more detailed description of the SPI algorithm is given elsewhere [50], [71]–[73].

### Household Climate Perceptions and Risks

Household surveys were used to assess local farmers' perceptions of environmental change near Kibale. Two research areas were defined within 5 km of the park boundary on the east and west sides of the park. The two regions differ in altitude, ethnic composition, and settlement and land use history. The east study area (56 km^2^) is settled predominately by Bakiga households, while the west study area (110 km^2^) is settled predominately by Batoro households (Fig. 1). A set of 95 random geographic coordinates within these areas was selected, and those points became the centers of 9-hectare areas (circles with radii of 170 m) termed “superpixels” (black circles in Fig. 1) [78]. Interview respondents were selected in each superpixel for which there were landholders (n = 68, 36 on the west side and 32 on the east side). The number of respondents selected per superpixel was proportional to the number of landholders controlling land within the superpixel and at least one interview was conducted in each superpixel. Houses were selected based on proximity to the center of the superpixel. The closest house was selected for the first interview, the next closest for the second interview, and so on. A full description of the geographic selection methodology can be found in Hartter (2009) [46].

Three separate surveys were conducted in 2005, 2006, and 2009, all of which used the superpixel sampling framework. Some of the same individuals or households were interviewed in multiple surveys.

1. *2005/6 survey (n = 70):* Between May and June 2005 and 2006, respondents were asked general questions about household composition, employment, and land use; and then using participatory risk mapping, respondents were asked to identify (free-list) any risks they and/or their families had [79], [80]. Respondents then ranked the risks in order of importance to the household.
2. *2006 survey (n = 130):* Between May and August 2006, respondents were asked about land use, forest fragment and wetland use, crop raiding, and their impressions of Kibale National Park. Additional results of these surveys are described elsewhere [2], [46], [60].
3. *2009 survey (n = 100):* Between May and August 2009, respondents were asked about perceived changes to the local climate, which are described in Kirner (2010) [81].

Interviews were conducted in person by Hartter, Goldman, and/or Kirner using a trained local interpreter in one of the main local languages, Rutoro or Rukiga, or in English. Questions were mostly open-ended, and respondents could further expound on their initial responses. Responses were then coded into categories during data analysis. Relationships between categorical responses and independent variables were examined with chi-square tests for independence (gender, location, wealth, newcomer status), while continuous variables were examined using Mann-Whitney U-tests (residence time, distance to park, and respondent age).

## Results

### Season Onset, Cessation, Duration, and Total Rainfall

**Figure 2:**
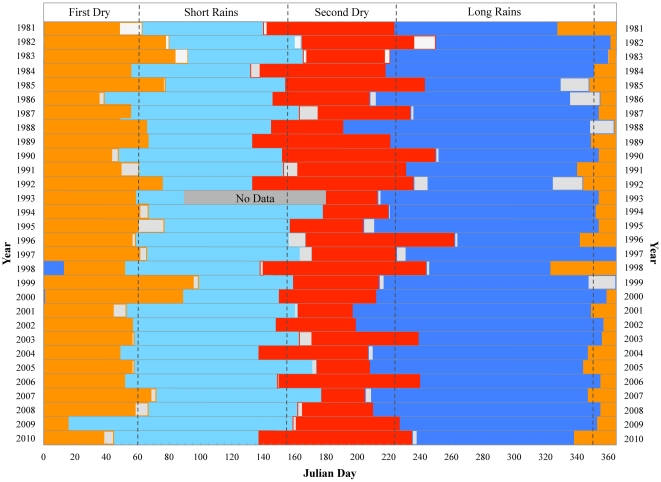
Distribution of rainfall seasons at MUBFS, Kibale National Park for the climate normal period 1981 to 2010. The inter-annual distribution of rainfall seasons from total daily rainfall observations at MUBFS, 1981–2010. The light gray areas indicate the difference between predicted and observed season onset dates. The vertical dashed lines represent the average season onset for the period of record. The uncertainty (the difference between the start date observed and the start date predicted using a statistical model) in season onset for 1993 is not displayed due to missing data for April, May and June.

Timing and distribution of daily rainfall during the transition from one season to the next varied from year to year. Standard deviations (*σ*) about the mean season onset date for all seasons ranged between 10 to 20 days (Table 1) with a difference in season onset from one year to the next as high as 30 days. Absolute differences between observed and predicted season onset of less than five days occurred for seasons in which there was an abrupt transition from one season to the next. Differences as large as ±20 days occurred for a few seasons in which the transition was gradual (Fig. 2). However, based on the average and absolute difference between observed and predicted onset dates determined from consecutive no-rain day intervals, the predicted dates reproduced the observed inter-annual variability in season onset reasonably well.

**Table 1:** Observed (*predicted*) mean (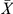), standard deviation (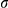), mean absolute error (MAE) and Pearson product moment correlation coefficient (*r*) for seasonal rainfall variables derived from daily rainfall observations at MUBFS for the period 1981–2010.

	ONS	DUR (days)	*P* (mm)	RD (%)	INT (mm day^−1^)
(A) First Dry^1^					
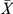	17 December	73	103.17	16.5	8.25
	(*13 December*)	(*80*)	(*135.54*)	(*18.3*)	(*8.83*)
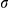	10.8 days	21.53	74.83	5.8	3.45
	(*11.9 days*)	(*22.1*)	(*86.52*)	(*6.0*)	(*3.43*)
MAE	3.2 days	8.2	36.65	2.9	1.14
*R*	0.84	0.91	0.82	0.67	0.87
(B) Short Rains^2^					
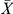	28 February	95	535.26	51.5	11.12
	(*3 March*)	(*91*)	(*512.86*)	(*51.4*)	(*11.16*)
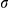	16.4 days	20.7	141.28	7.4	2.29
	(*16.6 days*)	(*20.6*)	(*142.23*)	(*7.8*)	(*2.49*)
MAE	3.8 days	6.0	23.78	1.3	0.31
*R*	0.96	0.95	0.98	0.97	0.99
(C) Second Dry^2^					
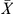	3 June	68	111.63	22.6	7.12
	(*3 June*)	(*69*)	(*121.93*)	(*21.8*)	(*7.44*)
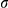	13.3 days	21.8	81.52	14.9	3.28
	(*31.2 days*)	(*22.9*)	(*95.02*)	(*7.6*)	(*3.35*)
MAE	2.5 days	4.9	16.15	3.4	0.98
*R*	0.94	0.93	0.96	0.74	0.87
(D) Long Rains					
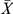	10 August	129	923.11	59.9	12.37
	(*12 August*)	(*124*)	(*877.35*)	(*60.1*)	(*12.34*)
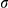	17.4 days	22.8	173.27	9.0	2.81
	(*18.0 days*)	(*24.7*)	(*160.34*)	(*9.8*)	(*2.72*)
MAE	2.3 days	7.1	46.97	1.2	0.30
*R*	0.98	0.91	0.94	0.97	0.99

1 The first dry season begins in December of the previous year and continues into February of the current year.

2 First rain and second dry season statistics are omitted for 1993 due to missing data for March, April and June of 1993.

The average absolute difference between a set of predicted (*p_i_*) and observed (*o_i_*) values, or the mean absolute error (MAE), was calculated to evaluate the predicted values [82]:
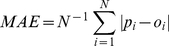
(1)Results (Table 1) show that the MAE was within one standard deviation of the mean onset date for all seasons. Therefore, any differences or prediction errors between the predicted and observed onset dates are within the range of natural variability. Furthermore, no prediction errors were significant (95% c.l.), indicating that the criteria used to predict season onset from daily rainfall observations provides a reasonable approximation of reality, and its application here is appropriate.

### Inter-Annual and Intra-Seasonal Variability in Rainfall

Significant trends in season rainfall variables occurred during the first dry, short rains and second dry seasons (Table 2). The only significant trends (90% c.l.) in season ONS and CES were associated with changes in the short rains. Significant inter-annual trends (95% c.l.) occurred in the DUR of the first dry and short rains, RD for the short rains and second dry, and *P* during the second dry. Significant intra-seasonal trends also occurred in the number and length of dry periods, defined as two or more consecutive days without rain, during the short rains (Table 3). Intra-seasonal distribution of rainfall during the long rains did not change significantly over the period of record.

**Table 2:** Seasonal time series trend statistics for MUBFS daily rainfall observations over the period of record 1981–2010.

	First Dry^1^		Short Rains^2^		Second Dry^2^		Long Rains	
	Trend	*p*-value	Trend	*p*-value	Trend	*p*-value	Trend	*p*-value
ONS (days)	4	0.30	*−14*	*0.08*	*12*	*0.08*	−5	0.31
CES (days)	*−14*	*0.07*	*12*	*0.07*	−6	0.30	6	0.18
DUR (days)	**−22**	**0.03**	**27**	**0.02**	−16	0.12	6	0.33
*P* (mm)	13.27	0.39	102.85	0.12	**−93.34**	**0.03**	−27.73	0.40
RD (%)	−0.5	0.45	**−11.2**	**0.01**	**−21.0**	**0.01**	−3.1	0.30
INT (mm day^−1^)	2.35	0.16	1.47	0.15	−1.44	0.23	0.13	0.47

Significant trends at the 90% c.l. are shown in italics and 95% c.l. are shown in bold.

**Table 3:** Average, standard deviation (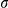) and trend in the number of two or more consecutive no-rain days and the mean, median and maximum length of no-rain periods within the short rains.

	Number	Mean (days)	Median (days)	Maximum (days)
Average	18	2	1.4	12
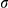	5	0.5	0.5	2
Trend	5.7	0.74	1.1	3.0
*p*-value	0.03	0.02	0.01	0.03

All statistics are significant at the 95% c.l. Short rains season statistics for 1993 are missing.

The significant trend toward and earlier ONS and later CES during the short rains accounts for the significant increase in the DUR of the short rains by 27 from 1981 to 2010 as well as trends in an earlier first dry CES and a later second dry ONS. The increase in DUR is negatively correlated with the significant, 11% decrease in the ratio of rain to no rain days (*r* = 0.30, *p*-value = 0.060) in favor of more days without rain. Total rainfall during the short rains is significantly correlated to DUR (*r* = 0.75, *p*-value<0.001), ONS (*r* = −0.59, *p*-value<0.001), and CES (*r* = 0.43, *p*-value = 0.010). Therefore, an increase in DUR due to an earlier ONS and later CES resulted in an increase in *P*, though this increase was not statistically significant.

As the proportion of days with rain during the short rains decreased, there was no significant change in INT, which increases as *P* increases (*r* = 0.60, *p*-value<0.001) and/or percent RD decreases (*r* = −0.44, *p*-value = 0.008). Analysis of the intra-seasonal distribution of rain and no rain days indicated that the number and length of dry periods during the short rains increased significantly (Table 3). Therefore, the decrease in rain days within the season does not result in a decrease in total seasonal rainfall. Rather, the rainfall occurs on fewer days within the season.

There was an overall decrease in rainfall during the second dry season with significant (95% c.l.) decreases in RD and *P*. Significant (95% c.l.) correlations exist between *P* and percent RD (*r* = 0.36, *p*-value = 0.027) and INT (*r* = 0.74, *p*-value<0.001) meaning that the second dry season is becoming drier overall with less rain falling over fewer days. Although not significant, there was a trend (−5.7 days) toward an earlier cessation of the second dry season from 1981 to 2010. Given the significant (95% c.l.) correlations between *P* and CES (*r* = 0.83, *p*-value<0.001) and DUR (*r* = 0.74, *p*-value<0.001), a shorter season due to an earlier end typically results in a decrease in *P*. The CES is correlated with INT (*r* = 0.59, *p*-value<0.001) and DUR (*r* = 0.82, *p*-value = 0.001), indicating a greater change in daily rainfall patterns toward the end of season transition to the long rains. This could be indicative of changing weather patterns during the long rains onset.

### Standardized Precipitation Index

**Figure 3:**
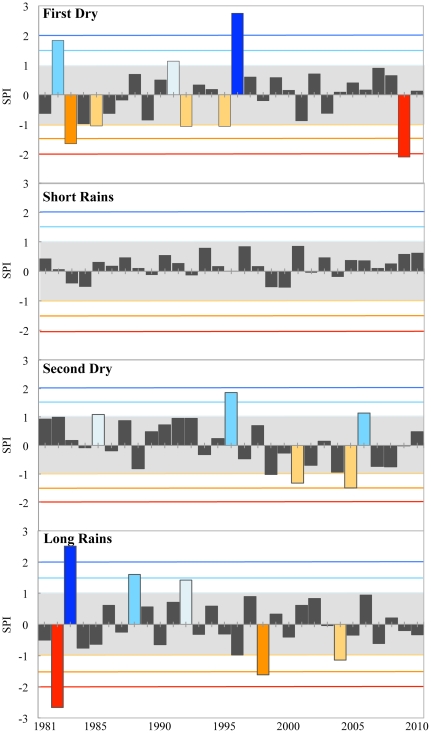
Time series of seasonal SPI values derived from MUBFS daily rainfall observations for the climate normal period 1981–2010.

The magnitude and direction of SPI values varied from year to year and season to season with climatological means within the range of normal variability (−1.0<SPI<1.0; Fig. 3). Significant trends toward higher, positive SPI values exist within the first dry (trend = 0.37; 95% c.l.) and short rains (trend = 0.28; 90% c.l.) season statistics with a significant trend toward abnormally dry conditions within the second dry season (trend = −1.14; 95% c.l.). While there was no significant trend in SPI values for the long rains, there was a significant decrease (trend = −0.89; 95% c.l.) in the absolute SPI value for the long rains. This indicates that the inter-annual variability in *P* decreased from 1981 to 2010 while trending toward more “normal” conditions.

The first dry season varied more than two standard deviations from the mean in 1996 (SPI = 2.75; extremely wet) and in 2009 (SPI = −2.10; extremely dry) (Fig. 3). Though trending toward positive SPI values, there was very little variability and no seasonal extremes during the short rains for all years. From 1981 to 2010, SPI values for the short rains remained near the seasonal mean and within the normal range of variability about the seasonal mean (Fig. 3). The second dry season did not exceed two standard deviations from the mean over the period of record, exceeding 1.5 standard deviations above the mean in 1996 (SPI = 1.86; severely wet) and one standard deviation from the mean in 2001 (SPI = −1.32; moderately dry), 2005 (SPI = −1.49; moderately dry), and 2006 (SPI = 1.14; moderately wet) (Fig. 3). Despite a “moderately wet” 2006 season, there has been a trend in the second dry season toward “moderately dry” conditions (SPI<−1.0) since 1981. The long rains varied more than two standard deviations from the mean in 1982 (SPI = −2.66; extremely dry) and 1983 (SPI = 2.51; severely wet). However, the magnitude of *P* extremes has decreased, ranging between normal (±1) and extremely abnormal (±2) in 1988 (SPI = 1.60; severely wet), 1992 (SPI = 1.42; moderately wet), 1998 (SPI = −1.62; severely dry), and 2004 (SPI = −1.15; moderately dry) (Fig. 3).

### Household Climate Perceptions and Risks

Climate variability or change is one of the substantive risks farmers face, though it usually is perceived as less widespread or significant than illness or crop raiding (although raiding is strongly affected by location with respect to the park or other areas that harbor crop-raiding animals). From the 2005/6 risk survey (n = 69), most respondents cited drought (80%) and excess rainfall (timing and/or amount) (54%) as among the risks they face. It is important to note that the local translation for drought is Ekyanda (in Rukiga) and Enjaara (in Rutoro), which means a prolonged period without rain and leading to a food shortage. This period is outside the “normal” dry seasons -meaning that if there is a prolonged dry season, whereby it would be dry in a time that would “normally” be the rainy season. Since this paper discusses perceptions of people, we did not want to constrain the definition of drought. (Other risks named included sickness 88% and crop raiding 42%). When asked to rank their risks, most respondents ranked sickness as a top-3 risk (83%), then drought (55%) and crop raiding (49%). Too much rain was ranked by 16% of respondents as a top-3 risk.

Our data from the 2009 survey indicate that 96% of the local farmers (n = 100) perceived that the timing and/or amount of seasonal rainfall had changed, while four respondents said there was no change. This perceived change in seasonal rainfall is widespread across the survey regions and does not appear to be a function of location or demographic characteristics (*p*-value>0.05) (Table 4). More than half of respondents (59%) reported that the timing of rainy season onset and/or cessation has become less predictable in recent years. Many respondents (43%) reported less total annual rainfall, while only 2% reported more rainfall overall. This perception was not affected by wealth or gender. However, older residents were more likely to report that the rains have changed (*p*-value = 0.015). More residents on the west side of Kibale (mainly Batoro) than those on the east side (mainly Bakiga) (*p*-value = 0.003) reported changes in season onset and cessation, but more east side residents reported less rain (*p*-value<0.001).

**Table 4:** Perceptions of local households of rainfall variability, change in season onset/session and less rain in recent years.

	Total n	Rains have changed	Season Onset/Cessation	Less Rain
Total	100	96%	59%	43%
Gender				
Male	34	97%	56%	47%
Female	66	95%	61%	41%
*p-value*		0.454	0.625	0.565
Side				
East	45	96%	44%	62%
West	55	96%	71%	27%
*p-value*		0.718	0.003	<0.001
Wealth				
Below average	16	94%	44%	63%
Average	79	96%	61%	42%
Above average	5	100%	80%	0%
*p-value*		0.303	0.436	0.121
Newcomer (< = 5 yrs)	17	88%	41%	53%
*p-value*		0.611	0.184	0.552
residence (total yrs/age)				
*p-value*		0.185	0.785	0.744
Distance to park				
*p-value*		0.246	0.136	0.562
Age				
*p-value*		0.015	0.314	0.531

*Gender*, *Side*, *Wealth*, and *Newcomer* tested using Pearson chi-squared analysis; *Residence*, *Distance to Park*, and *Age* tested using Mann-Whitney U-test. (2009 survey, n = 100).

Newcomer = respondent who came to the area within the last 5 years.

Residence = proportion of respondent's life at current farm.

Side = east or west side of Kibale National Park.

Weather plays an important role in daily life and is a common topic of conversation. Along with the widespread perception that weather patterns have changed or are highly variable, there is a general consensus among farmers on how to determine rainy season onset and cessation. Rainy season onset, as described by local farmers, occurs when sky cover changes to “heavy nimbus” (or rain) clouds, thunderstorms, and cool winds within the months that rain is expected. It ends two to three months later when cloud cover decreases and skies become clear with ample sunshine, the cloudless days become hot, and there are strong, dry winds. Farmers communicate these observations and interpretations to other farmers, which likely plays a role in influencing planting and harvesting decisions.

With over 95% of the respondents supporting their livelihoods as farmers, the timing and amount of seasonal rainfall have direct impacts on household food security. Seventy-six percent of respondents in the 2009 survey (n = 100) believe that changes in weather have affected their agricultural outputs. Unlike the perceptions of a changing climate, these perceptions are common throughout the landscape and do not differ by wealth, gender, newcomer status, or location (east or west side of park (*p*-value>0.05).

Farmers' responses suggest that they have observed impacts of land use change with respect to the park as well as intact forest fragments and wetlands in the domesticated landscape. Further, farmers identified non-material benefits from the park and natural areas, which in turn link to local climate. They report that the presence of forests from Kibale, and also the unprotected, small interstitial forests and papyrus wetlands outside the park, provided what could be characterized as ecosystem services. Perceived benefits from Kibale that were collapsed in to the “ecosystem services” category included: [Kibale] regulates climate, provides a moderate climate, provides rainfall (both timing and amount were mentioned), provides fresh air, provides cool air, provides habitat for wildlife, and maintains soil moisture near boundary. Again, these are respondents' own perceptions about benefits from Kibale. Rainfall and “fresh air”, attributed to the existence of these natural areas and Kibale, were most often mentioned (Table 5). Perception about “regulation of local climate” was mentioned quite often. Local residents described this as the maintenance of local weather conditions in general that were hospitable to their way of life. They tell us that without Kibale, this would be a dry area, it would be hot, and the land would not be suitable for farming (2006 survey, [83]). To them, the climate is more moderate because of the presence of Kibale and these natural areas.

**Table 5:** Farmers' perceptions that forest fragments, wetlands, and Kibale National Park provide ecosystem services.

Ecosystem services	Forests (outside park)	n	Park	n	Wetlands (outside park)	N
Ecosystem services	16%	21	43%	52	31%	40
Rain (timing & amount)	14%	18	36%	47	23%	30
Fresh air	9%	12	14%	18	8%	11
Regulation of local climate	1%	1	14%	18	3%	4
Soil moisture	3%	4	2%	2	6%	8
Soil fertility	0%	0	2%	2	0%	0

(2006 survey, n = 130).

## Discussion

Many African countries are vulnerable to climate variability and change, in part because they have only a limited capacity to adapt to changing circumstances [84]. A high reliance on natural resources, high poverty, limited capital to invest in mitigation and adaptation strategies, and inadequate institutional capacity means that any environmental changes affecting resource availability will result in hardship [84]. In Uganda, where rain-fed agriculture constitutes 42% of the gross domestic product and over 90% of the export earnings [85], sustainable livelihoods are directly related to food security. Objective, quantitative information on seasonal rainfall variability and trends is crucial for timely implementation of sustainable agricultural practices to deal with present and predicted change.

Analyses of other regions in Africa [57] indicate an increase in drought conditions over much of the continent; however these tend to be at a coarse scale and focus on rain-poor regions (e.g., savannas in East Africa) [48], [55]. Increasing drought frequency in East Africa and Uganda [86] tends to be restricted geographically to the drier regions of Uganda north and northeast of our study area. In contrast, even with some variability, Kibale has adequate rainfall with few significant trends in total seasonal rainfall, season onset and cessation. Climatologically, this is an area of high rainfall with a bimodal distribution.

Perceptions of local farmers are important because farmers often manage land according to their perceptions and beliefs [87]. In these communities, meteorological information from the scientific community is rarely available, and farmers rely on their own observations and subjective interpretations. Despite the fact that this is an area of high rainfall, “drought” – which usually comprises insufficient rain at critical periods in the agricultural calendar – is frequently cited as an important risk, as is excess rain. When we quantified changes in direction and magnitude of seasonal rainfall, overall it appeared not to validate local perceptions. However, a closer examination of the rainy seasons revealed dynamics that may be contributing to local perceptions of altered seasonal timing.

Our analyses indicate that despite fairly consistent seasonal rainfall totals, the short rains have had more no rain days since the mid-1990s with longer periods of no rain days in between rain events. The second dry season has become drier, and there have been fewer abnormally wet long rains since 1981. In the absence of instrumental rainfall records, these weather patterns may manifest in perceptions of a local “drying.” In addition, people may base their responses on extreme conditions or events that caused the greatest hardships [56]. Many respondents believed that the timing and duration of the rainy seasons has varied. They report that they cannot depend on the timing and amount of rainy season precipitation, as it has become unpredictable compared to the past. We found that there was high inter-annual variability in season onset and cessation over the period of record for all seasons and the transition from the first dry season to the short rains has become less distinct. This variable season onset and change in transition between dry and rain seasons may lead farmers to perceive changing seasonal timing, which may have substantial impacts on crop planting and harvesting.

Many communities near Kibale fear changes in seasonal rainfall amount and duration, but few households distinguish general (aggregate) trends from seasonal trends. Peoples' comments suggest they blended the seasons and years together to form their own perceptions of rainfall variability. Overall, local farmers' perceptions of changing rainfall are more extreme than the rainfall data suggest. However, it is important to be aware of these perceptions since people frequently act on their perceptions, change their behavior, and develop coping strategies based on their dynamic and evolving knowledge, whether or not they are consistent with meteorological data [88], [89].

Determining the start, end, and duration of the rains is important to people. Local farmers explain simply that planting and harvesting cycles begin and end when the rainy season comes and goes, and when crops are ripened to maturity. Given the variability in season onset and the amount that farmers perceive and experience, many report that it is difficult to determine when to plant and harvest. Heavy rainstorms, short heavy rains, or extended periods of no rain during the rainy season can affect productivity and agricultural activities. Harvesting crops too soon or too late has implications for food security in the short-term (food for the family), and long-term, since farmers need a seed source for the following season. Around Kibale, many people are worried about drought, and most respondents reported experiencing decreased agricultural outputs.

Perceptions based on individual and collective interpretations are likely shaped by a number of interacting factors, such as access to information, formal education, social interactions, and life experience [90]. We were unable to detect significance for these variables in our analysis; however, they are likely still contributing to overall perceptions. For example, older people may be more likely to report changes in rain because they have had more experience on the land and with farming – i.e., a larger proportion of their life has been tied to the outputs of rain-fed agriculture. Therefore, they may draw their impressions from a much longer temporal scale. Farmers around Kibale have been reporting increased inter- and intra-annual and seasonal variability since at least the 1970s, (T. Struhsaker, personal communication). Such interpretations of long-term average environmental conditions tend to be influenced by recent, short-term weather events as well as memories of extreme events, such as drought and periods of food insecurity. These impressions tend to be stronger than those from periods of normal conditions and help to shape judgment and comparisons to successive events or seasons [56], [91], [92]. We found that more farmers on the east side versus the west side of Kibale report less rainfall than in the past. Inadequate rainfall may be more noticeable to east side residents because maize is more commonly planted on this side (bananas are much more common on the west side). Since maize is planted seasonally, whereas bananas are planted annually, the effects of “less rainfall” may be more evident in maize. Clearly, this calls for a more detailed survey to understand the nuances of indigenous knowledge about climate change in this area. It is likely that the perceived decrease in agricultural output is indicative of broader-scale landscape changes. Mid- to high-altitude tropical forest areas with good to very good soils, like Kibale, have unusually high agricultural potential (as compared to the far more extensive lowland forests with highly aged soils); and thus support high-density populations and small-scale agriculture, putting enormous resource pressures on protected and unprotected forests [21], [22], [57], [87]. This ability to support particularly high densities of small-scale agriculture may result in highly amplified impacts of change. This suggests that there is a high potential for climate and land use dynamics to exacerbate park vulnerability to resource exploitation. Climate change is expected to lead to drastic shifts of biodiversity-rich biomes [22], and these changes are expected to be particularly profound in tropical forests where the highest concentrations of biodiversity and endemism are found. Increased population density leads to increased land conversion and land use intensification surrounding parks, which changes ecological function and biodiversity within parks [6], and also increases pressure for access to resources in the park. Changes in food abundance within parks may also force wildlife to seek food in agricultural areas near the park boundary, increasing the vulnerability of farms to crop damage and predation [45], [60].

Stampone et al. [50] have shown that the Kibale region has high spatial variability in rainfall due to topography and other factors, and the discrepancy between perception and rainfall trends points toward the need for better information on seasonal and annual rainfall patterns at the local level [60]. There is, therefore, a strong need to educate local people and conservation managers alike using a whole landscape approach [65], [93], [94] to climate change mitigation and adaptation that includes both the park and surrounding domesticated landscape. Results of this research will provide local people with more relevant and physically accurate information that, if accepted, could lead to more sustainable land use management practices outside the park that are concurrent with conservation objectives.
